# Postoperative abdominal sepsis induces selective and persistent changes in CTCF binding within the MHC-II region of human monocytes

**DOI:** 10.1371/journal.pone.0250818

**Published:** 2021-05-03

**Authors:** Benedikt Hermann Siegler, Marc Altvater, Jan Niklas Thon, Christopher Neuhaus, Christoph Arens, Florian Uhle, Christoph Lichtenstern, Markus Alexander Weigand, Sebastian Weiterer

**Affiliations:** 1 Department of Anesthesiology, Heidelberg University Hospital, Heidelberg, Baden-Württemberg, Germany; 2 Department of Anesthesiology, Rheinland Klinikum Neuss, Lukaskrankenhaus, Neuss, Nordrhein-Westfalen, Germany; University of California San Francisco, UNITED STATES

## Abstract

**Background:**

Postoperative abdominal infections belong to the most common triggers of sepsis and septic shock in intensive care units worldwide. While monocytes play a central role in mediating the initial host response to infections, sepsis-induced immune dysregulation is characterized by a defective antigen presentation to T-cells via loss of Major Histocompatibility Complex Class II DR (HLA-DR) surface expression. Here, we hypothesized a sepsis-induced differential occupancy of the CCCTC-Binding Factor (CTCF), an architectural protein and superordinate regulator of transcription, inside the Major Histocompatibility Complex Class II (MHC-II) region in patients with postoperative sepsis, contributing to an altered monocytic transcriptional response during critical illness.

**Results:**

Compared to a matched surgical control cohort, postoperative sepsis was associated with selective and enduring increase in CTCF binding within the MHC-II. In detail, increased CTCF binding was detected at four sites adjacent to classical HLA class II genes coding for proteins expressed on monocyte surface. Gene expression analysis revealed a sepsis-associated decreased transcription of (i) the classical HLA genes *HLA-DRA*, *HLA-DRB1*, *HLA-DPA1* and *HLA-DPB1* and (ii) the gene of the MHC-II master regulator, CIITA (Class II Major Histocompatibility Complex Transactivator). Increased CTCF binding persisted in all sepsis patients, while transcriptional recovery CIITA was exclusively found in long-term survivors.

**Conclusion:**

Our experiments demonstrate differential and persisting alterations of CTCF occupancy within the MHC-II, accompanied by selective changes in the expression of spatially related HLA class II genes, indicating an important role of CTCF in modulating the transcriptional response of immunocompromised human monocytes during critical illness.

## Introduction

Postoperative abdominal infections rank among the most common causes of sepsis and septic shock in intensive care units (ICU) worldwide [1], associated with detrimental short- and long-term outcomes [2]. During sepsis—defined as life-threatening organ dysfunction based on a dysregulated host response to pathogens [3]—apoptosis and functional impairment of antigen-presenting cells contribute to a persisting state of immune paralysis with a high risk of opportunistic infections and death [4]. The underlying pathophysiology is characterized by a decline in monocyte surface expression of the human leucocyte antigene DR (HLA-DR), an independent predictor of mortality in critically ill patients [5–7]. While monocytes play a central role in mediating the initial host immune response during sepsis [8], low HLA-DR expression levels have been linked to monocyte anergy, including a decrease of cytokine production following microbial challenges as well as defective antigen presentation via HLA class II proteins [9, 10].

The genetic information for proteins involved in antigen presentation is located in the MHC region on the short arm of human chromosome 6 (6p21.3) [11]. Within the extended MHC, MHC-II contains several genes encoding for alpha- and beta-chain components of the classical isotypes HLA-DP, HLA-DQ and HLA-DR that are involved in monocyte surface presentation of microbial peptides to CD4^+^-T-lymphocytes [12]. Besides, the almost 700 kb covering region hosts genes for the heterodimeric molecules HLA-DO and HLA-DM, both assisting the process of antigen presentation [13], as well as non-HLA proteins with other functions including Transporter associated with Antigen Processing 1 and 2 (TAP1 and TAP2), Proteasome subunit beta type-8 and -9 (PSMB8 and PSMB9) and the Bromodomain-containing protein 2 (BRD2) [11].

Transcriptional regulation of the MHC-II is at least in part controlled by the Class II Major Histocompatibility Complex Transactivator (CIITA) and its interplay with distinct DNA-binding transcription factors and cofactors, resulting in the assembly of a heteromultimeric enhanceosome complex [14, 15]. Cell culture experiments revealed that this complex interacts with the zinc-finger protein CCCTC-Binding Factor (CTCF) in a region separating the genes *HLA-DRB1* and *HLA-DQA1*, which seems to be responsible for the changes in the investigated HLA class II gene regulation [16, 17]. Recently, we could demonstrate in circulating monocytes of patients with sepsis, that an increased CTCF-occupancy at this intergenic region is associated with selective changes in adjacent HLA-gene expression. These findings indicate a CTCF mediated enhancer blockade as a potential mechanism contributing to an immunocompromised phenotype [18].

So far, approximately 20.000 to 40.000 CTCF-binding sites have been discovered in the human genome [19–21], including ten within the MHC-II [22]. Since CTCF is a globally occurring DNA-binding protein with multiple functions including the organisation of higher-order chromatin architecture, insulation of transcriptionally active regions and the blockade of enhancers [23], we investigated sepsis-induced changes in CTCF binding inside the entire MHC-II region with impact on HLA gene transcription.

## Results

### Study cohort

**Fig 1:**
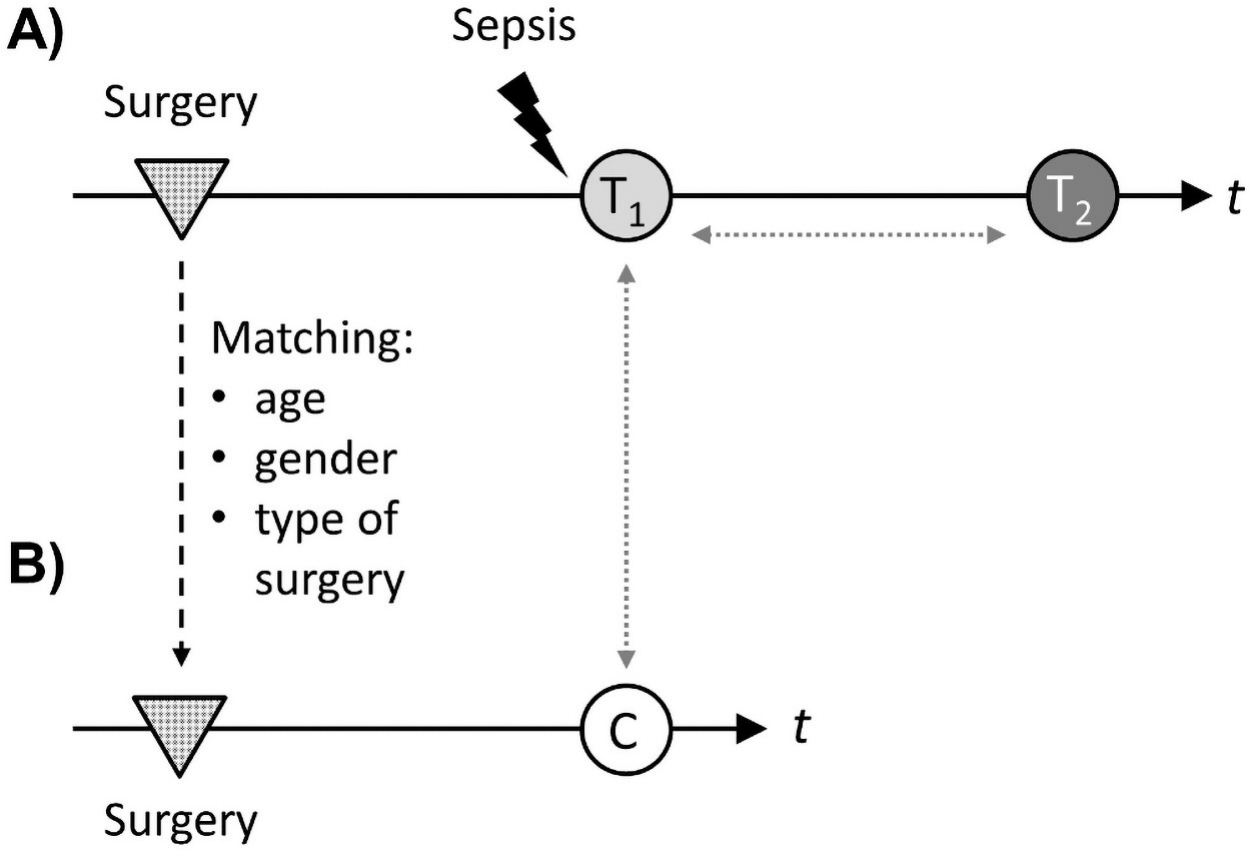
Study design. **(A)** Nine patients with sepsis after major abdominal surgery were included. Blood samples for experimental workflow were taken within 24h after diagnosis of sepsis (light grey circle, “T1”) and 7 days thereafter (dark grey circle, “T2”). Time (t) between surgery (grey dotted triangles) and sepsis diagnosis was recorded. **(B)** Control patients were identified by matching for age, gender and type of surgery. Seven days after surgery, blood samples were taken for experimental workflow (white circle, “c”). Comparisons were performed between control patients and patients with sepsis at T1, as well as between T1 and T2 in the cohort of sepsis patients (grey dotted arrows).

Overall, 9 patients with sepsis after major abdominal surgery and 9 matched control patients were included (Fig 1).

Both groups consisted of 4 female and 5 male individuals with a median age of 72 years (range 50–78) among patients with sepsis and 69 years (range 51–79) in the group of surgical control patients (p = 0.85). No differences were found concerning comorbid conditions. Median sequential organ failure score (SOFA score) was 13 (range 10–17) among sepsis patients at the time of study inclusion. S1 Table summarizes clinical and socio-demographic characteristics of the investigated patient collectives.

### Sepsis-associated cellular response to inflammatory stimuli

**Fig 2:**
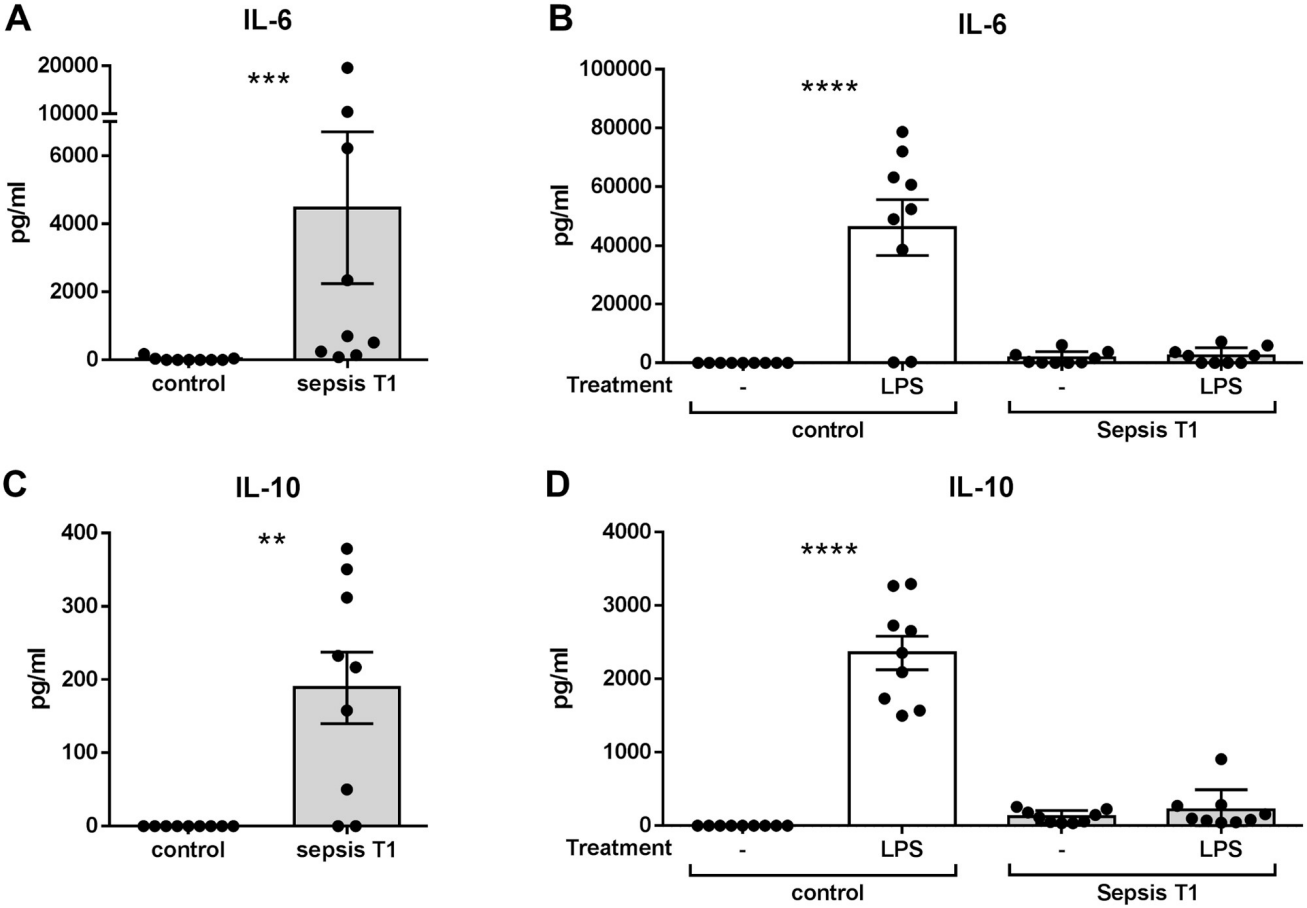
Cytokine levels in plasma and supernatants from ex-vivo stimulated samples. Plasma levels of pro- and anti-inflammatory cytokines from patients with sepsis at the time of sepsis diagnosis (T1) were compared with healthy control patients. IL-6 levels in **(A)** plasma (p<0.001, Mann-Whitney U test, control patients n = 9, patients with sepsis n = 9, mean ± SEM) and **(B)** in supernatants after ex-vivo stimulation with LPS (p<0.0001, Mann-Whitney U test, control patients n = 9, patients with sepsis n = 9, mean ± SEM). IL-10 levels in **(C)** plasma (p<0.01, Mann-Whitney U test, control patients n = 9, patients with sepsis n = 9, mean ± SEM) and **(D)** in supernatants after ex-vivo stimulation with LPS (p<0.0001, Mann-Whitney U test, control patients n = 9, patients with sepsis n = 9, mean ± SEM).

Sepsis-related immune alterations included changes in plasma levels of both pro- and anti-inflammatory cytokines. Compared to control, sepsis was associated with higher levels of IL-6 (median 0.0 pg/ml vs. 703 pg/ml; ***p<0.001) and IL-10 (median 0.0 pg/ml vs. 217 pg/ml; **p<0.01) at the time of study inclusion (Fig 2A and 2C). To assess the *ex vivo* cellular response to a secondary inflammatory stimulation, whole blood was incubated with the Toll-like receptor 4 agonist LPS. While levels of IL-6 and IL-10 strongly increased in control patients, patients with postoperative sepsis were unresponsive to LPS stimulation (Fig 2B and 2D), hinting towards sepsis-induced cellular anergy at the time of study inclusion.

### Sepsis leads to a decrease in monocytic HLA-DR expression

**Fig 3:**
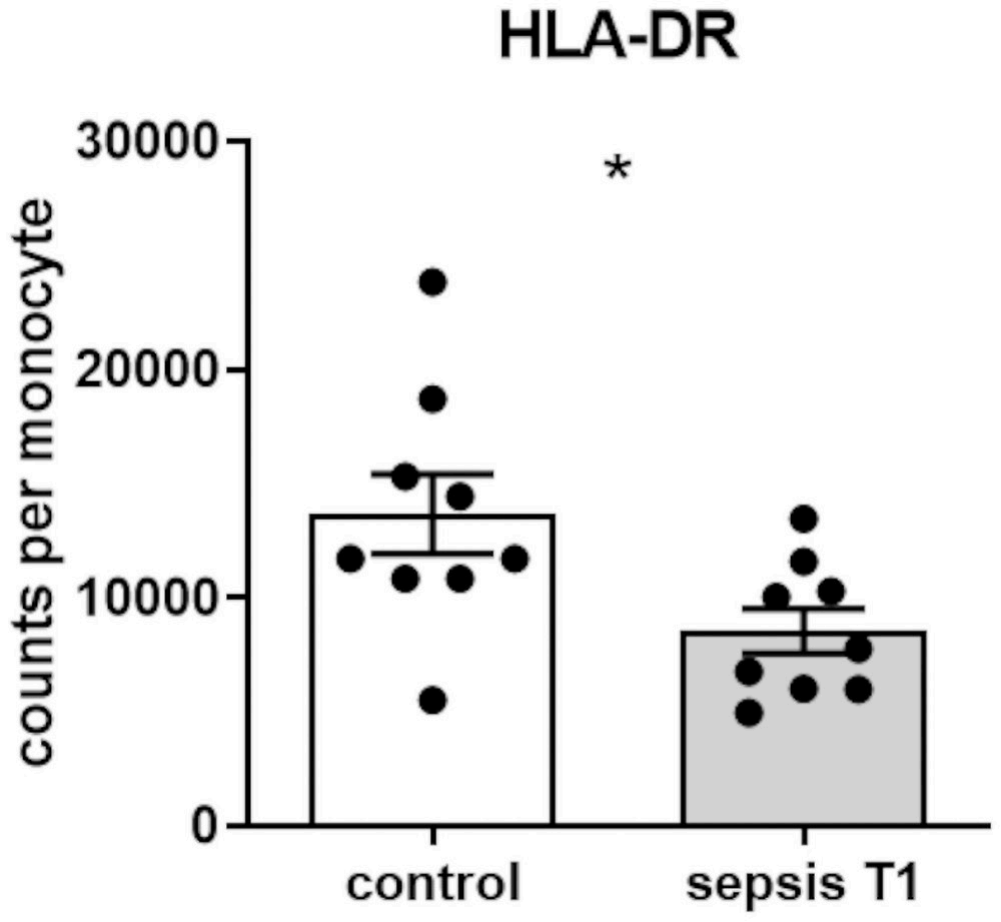
HLA-DR expression on monocyte surface. Surface expression of HLA-DR on CD14^++^-monocytes at the time of sepsis diagnosis (T1) compared to matched control patients analyzed in whole blood samples using flow cytometry (p*<*0.05, Mann-Whitney U test, control patients n = 9, patients with sepsis n = 9, mean ± SEM).

To confirm the observed immunocompromised phenotype, monocytic HLA-DR surface expression, a surrogate for immunosuppression in sepsis [24, 25], was analyzed via flow cytometry. As expected, patients with sepsis displayed reduced levels of monocytic HLA-DR surface expression compared to surgical control patients (median 7788 vs. 11727 molecules per monocyte, *p<0.05, Fig 3).

Low HLA-DR expression levels have not only been linked to monocyte anergy in terms of decreased cytokine production following microbial challenges, but also to alterations in antigen presentation via HLA class II proteins [9, 10]. Since the process of antigen presentation is mainly controlled on the level of gene transcription [14], further experiments focused on the MHC-II region containing genes encoding for chain components of receptor molecules involved in monocyte surface presentation of microbial peptides and its superordinate regulation.

### Sepsis decreases expression of the transcriptional co-activator CIITA and increases expression of the superordinate regulator CTCF

**Fig 4:**
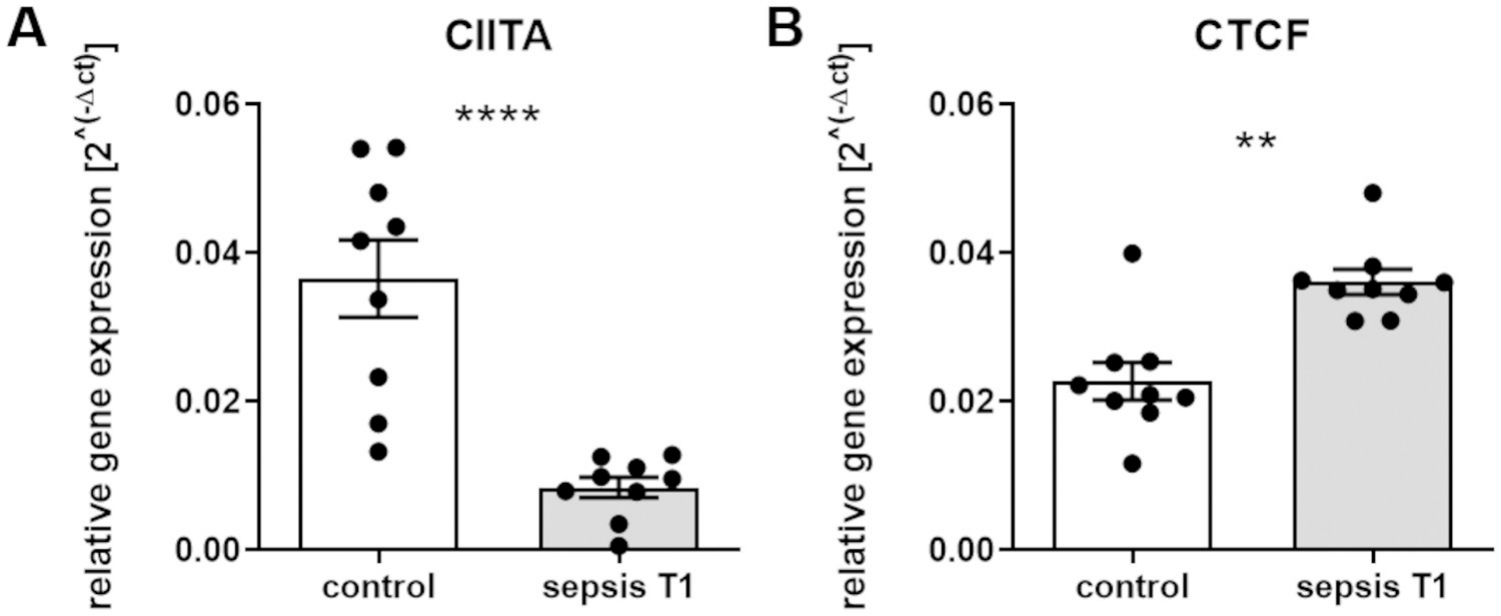
Expression of the transcriptional co-activator CIITA and the superordinate regulator protein CTCF. RNA from isolated human CD14^++^ monocytes was used for reverse transcription and subsequent qPCR using TaqMan Assays against **(A)** *CIITA* and **(B)** *CTCF*. Relative gene expression was compared between control patients and patients with sepsis at the time of sepsis diagnosis (T1; ****p*<*0.0001 (CIITA) and **p<0.01 (CTCF), Mann-Whitney U test, control patients n = 9, patients with sepsis n = 9, mean ± SEM).

Regulation of MHC-II has been shown to depend on the transcriptional co-activator CIITA and its interplay with distinct DNA-binding factors with subsequent formation of an enhanceosome complex [26]. In previous work, the *CIITA* promoter located on the short arm of chromosome 16 displayed a marked reduction of the histone marks H3K4me3 and H3K27ac in patients with sepsis compared to healthy control individuals [18, 27]. Because these modifications are predominantly associated with a higher transcriptional activity [28], RNA from circulating CD14^++^ monocytes was used for reverse transcription and subsequent qPCR using TaqMan Assays against *CIITA*. Patients with postoperative sepsis were characterized by a strong decline in *CIITA* mRNA expression compared to surgical control patients (****p<0.0001, control vs sepsis T1, Fig 4A). In addition, since CIITA has been shown to interact with the superordinate regulator protein CTCF [16, 17], expression of the *CTCF* gene was analyzed. Sepsis was associated with an increased *CTCF* expression (**p<0.01, control vs sepsis T1, Fig 4B). Moreover, an inverse correlation of *CTCF* and *CIITA* expression was detected in patients with postoperative sepsis (R^2^ = 0.48; p<0.05).

### Sepsis selectively increases CTCF occupancy within the MHC-II region

Cell culture experiments by Majumder and colleagues revealed that the enhanceosome complex interacts with CTCF within the MHC-II region in an intergenic region separating the genes *HLA-DRB1* and *HLA-DQA1*, which seems to be responsible for changes in HLA class II gene regulation [16, 17]. We could recently show that increased CTCF-occupancy at this intergenic region is associated with differential expression of spatially related genes in critically ill and immunocompromised patients [18]. To elucidate the overall CTCF occupancy within the MHC-II region and its potential impact on HLA gene expression during postoperative abdominal sepsis, we analyzed all known CTCF binding sites within MHC II as identified by Majumder and Boss [22]. To rule out potential effects of differential CTCF expression and to address assay background, chromatin from isolated circulating CD14^++^ monocytes was immunoprecipitated with anti-CTCF antibody for subsequent qPCR using primers against specific positive and negative control regions outside the investigated MHC subregion. No relevant CTCF-binding was observed inside the negative control region (Myoglobin, *MB*, located on chromosome 22) and neither *MB* nor *H19ICR* (H19/IGF2 Imprinting Control Region, positive control region located on chromosome 11) differed in CTCF occupancy comparing septic patients at T1 and T2 with the surgical control cohort (S1 Fig).

**Fig 5:**
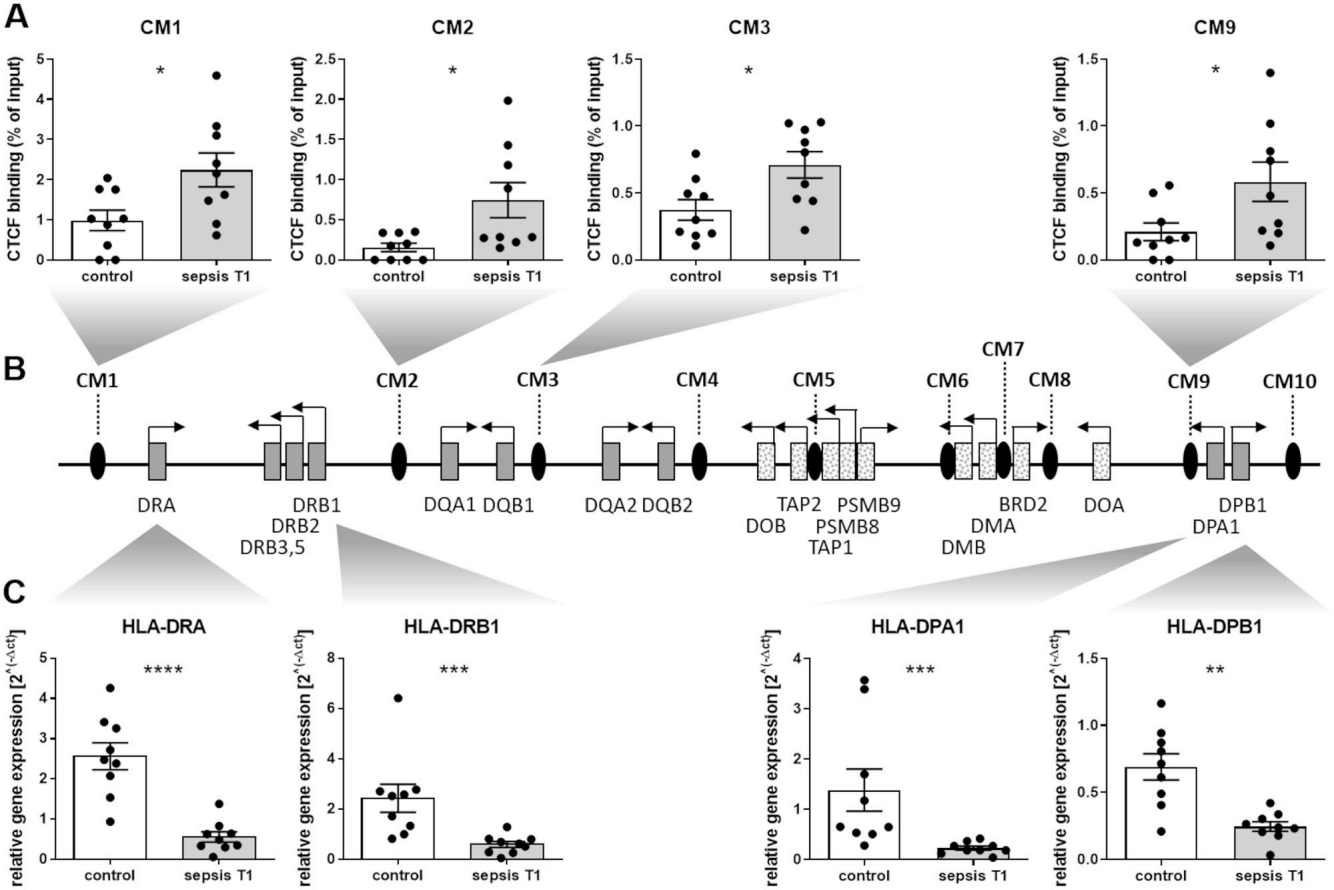
CTCF occupancy at specific binding sites within the MHC-II region and differential expression of classical HLA genes. **(A)** Chromatin from isolated human CD14^++^ monocytes (control patients and sepsis patients at the time of sepsis diagnosis, T1) was immunoprecipitated with anti-CTCF antibody for subsequent qPCR using primer pairs on specific target regions within the MHC-II region identified by Majumder and Boss [22]. Enrichment of CTCF at the binding sites CM1, CM2, CM3 and CM9 is shown (p*<*0.05, Mann-Whitney U test, control patients n = 9, patients with sepsis n = 9, mean ± SEM). **(B)** Schematic diagram of human chromosome 6p21.3 showing the relative positions of CTCF binding sites (black ovals) and MHC-II genes (boxes, classical HLA genes are shown as filled boxes). **(C)** RNA from isolated human CD14^+^ monocytes was used for reverse transcription and subsequent qPCR experiments using TaqMan Assay against *HLA-DRA*, *HLA-DRB1*, *HLA-DPA1* and *HLA-DPB1* (p<0.0001, p<0.001 and p*<*0.01, Mann-Whitney U test; control patients n = 9, patients with sepsis n = 9. Mean ± SEM).

Among putative CTCF sites sharing a consensus core sequence, Majumder and colleagues confirmed ten sites within MHC-II [22], which were termed CM1-10 in the present study. Using chromatin from isolated CD14^++^ monocytes, ChIP experiments and subsequent qPCR revealed a differential CTCF occupancy at the investigated sites during postoperative sepsis. In detail, sepsis was associated with a selective increase of CTCF enrichment at four binding sites that are located adjacent to the classical HLA gene isotypes HLA-DR, HLA-DQ and HLA-DP: CM1 (neighboring the *HLA-DRA* gene, *p<0.05), CM2 (located between the diametrically transcribed *HLA-DRB1* and *HLA-DQA1* genes, *p<0.05), CM3 (located between *HLA-DQB1* and *HLA-DQA2*, *p<0.05) and CM9 (spatially related to *HLA-DPA1* and *HLA-DPB1*, *p<0.05; Fig 5A and 5B).

In contrast, no differential CTCF occupancy was detected at the binding sites CM 4, 5, 6, 7, 8 and 10 (S2 Fig).

To determine whether histone acetylation could explain the observed variances in CTCF binding within the MHC-II region during sepsis, additional ChIP-experiments were performed using antibodies against H3K27ac, a mark associated with an open chromatin structure [28]. Assay background was addressed by anti-H3K27ac ChIP experiments and subsequent qPCR using primers against specific positive (*EIF4A2*, Eukaryotic Translation Initiation Factor 4A2, chromosome 3) and negative control regions (*MYT1*, Myelin Transcription Factor 1, chromosome 20) outside the investigated gene cluster (S1 Fig). Within the MHC-II region, a sepsis-associated increase in H3K27ac was found at CM1 (*p<0.05), whereas CM2, CM3 and CM9 did not differ in the level levels of histone acetylation (p>0.05, S3 Fig).

### Differential expression of classical HLA genes

Since the observed sepsis-associated increase of CTCF occupancy exclusively occurred at binding sites adjacent to classical HLA class II isotypes (HLA-DR, HLA-DQ and HLA-DP, Fig 5A and 5B), transcription of the corresponding genes was analysed. Interestingly, we found that the expression of HLA-DR- and HLA-DP-genes was differentially affected in patients with sepsis compared to control patients depending on the localization relative to the investigated CTCF-binding sites (Fig 5B and 5C). In detail, transcription of genes encoding for HLA-DR subunits (*HLA-DRA* and *HLA-DRB1*) was strongly reduced in patients with postoperative sepsis (****p<0.0001 and ***p<0.001). Both genes neighbour CTCF binding sites with an observed increase in CTCF occupancy during postoperative sepsis (CM1 and CM2). Further experiments showed a decreased expression of the HLA-DP genes *HLA-DPA1* and *HLA-DPB1* (***p<0.001 and **p<0.01), which are placed adjacent to the CTCF binding site CM9, Fig 5C). Remarkably, correlation analyses revealed that expression of HLA-DRA and HLA-DRB1, HLA-DPA1 and HLA-DPB1 correlated with both CIITA and CTCF expression (S2 Table).

In contrast, no differences were found in the transcription of *HLA-DRB3*, *HLA-DRB5* or the HLA-DQ subunits A1, B1, A2 and B2 (S4 Fig).

Next, we investigated whether the differential HLA gene expression was also accompanied by changes in H3K4me3, a mark known to be associated with open chromatin structure and transcription [28]. Anti-H3K4me3 ChIP experiments using primers against specific positive (*EIF4A2*, Eukaryotic Translation Initiation Factor 4A2, chromosome 3) and negative control regions (Myoglobin, *MB*, located on chromosome 22) outside the MHC-II region were conducted to address assay background. No relevant levels of H3K4me3 were observed inside the negative control region and neither positive nor negative control regions differed in H3K4me3 comparing patients with sepsis at T1 and T2 with the surgical control cohort (S1 Fig). Within MHC-II, we found overall moderate levels of H3K4me3 in the promoter regions of HLA-DRA and HLA-DPA1, compared to lower levels at HLA-DRB1 and HLA-DPB1, but could not detect significant differences between septic patients and individuals of the surgical control cohort (S5 Fig).

### Time course of sepsis-induced alterations and long-term survival

To elucidate whether the observed (epi)genetic alterations persevere as part of prolonged impairment of the immune system, additional blood samples were taken from patients with sepsis 7 days after the time of study inclusion (Fig 1). Laboratory parameters associated with sepsis as well as cytokine levels in plasma at both time points are summarized in S3 Table. Whereas lactate levels, which are associated with mortality in sepsis [29, 30], as well as plasma concentrations of the proinflammatory IL-6 significantly dropped comparing T1 with T2, patients with sepsis displayed persisting high levels of IL-10, accompanied by a sustained decline in monocyte HLA-DR surface expression (S3 Table and S6 Fig).

Additional experiments revealed that the observed elevation in *CTCF* expression, CTCF binding at CM1, CM2, CM3 and CM9 as well as the reduced expression of adjacent classical HLA genes *HLA-DRA*, *HLA-DRB1*, *HLA-DPA1* and *HLA-DPB1* persisted in all patients with postoperative sepsis (S7–S9 Figs).

**Fig 6:**
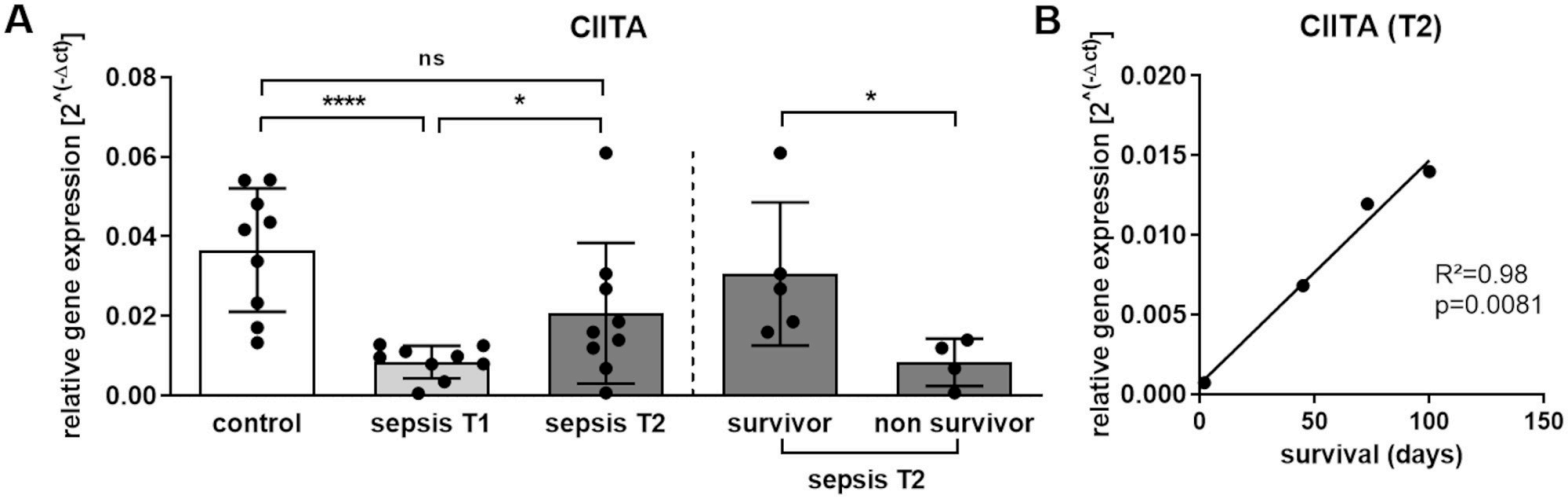
Expression of transcriptional co-activator CIITA gene, time course of sepsis-associated alterations and survival. **(A)** RNA from isolated human CD14^++^ monocytes was used for reverse transcription and subsequent qPCR using TaqMan Assay against *CIITA*. Relative *CIITA* expression was compared between control patients and patients with sepsis at the time of sepsis diagnosis (T1) and 7 days thereafter (T2). In the sepsis cohort, four patients died within one year (non-survivors); *CIITA* gene expression in these patients was further compared with *CIITA* expression in survivors at T2 (p<0.0001, Mann-Whitney U test, control patients n = 9, patients with n = 9, survivors n = 5, non-survivors n = 4, mean ± SEM). **(B)** Correlations between relative expression values of *CIITA* gene 7 days after study inclusion (T2) and the number of surviving days in patients who died within one year after sepsis diagnosis were analyzed (R^2^ = 0.98, p = 0.0081).

However interestingly, we found a recovery of *CIITA* transcription levels comparing septic patients at T1 and T2 (*p<0.05, left side of Fig 6A).

Four individuals of the sepsis cohort died within one year after study inclusion (see S1 Table). In all cases, abdominal sepsis was documented as cause of death. Neither monocytic HLA-DR surface expression, nor CTCF gene expression and its binding within the MHC-II region or HLA class II gene expression differed between sepsis survivors and non-survivors (S6–S9 Figs). However remarkably, non-survivors were characterized by a failure to reconstitute *CIITA* expression (*p<0.05; right side of Fig 6A). A Spearman correlation test used to determine whether *CIITA* transcription levels were linked to the number of surviving days, found a strong correlation between the expression of the master regulator and survival time (R square = 0.98, **p<0.01; Fig 6B).

## Discussion

The DNA-binding zinc finger protein CTCF has been identified as major transcriptional regulator involved in higher-order chromatin architecture, insulation of transcriptionally active regions and the modulation of enhancer function [31]. The impact of CTCF on global gene expression has been investigated in numerous cell types including those of the innate immune system [23]; however, its role in the transcriptional regulation of antigen presentation during postoperative human sepsis has not been elucidated so far. Based on previous work hinting towards a CTCF-mediated enhancer blockade of selected HLA genes during critical illness [18], we analysed global CTCF occupancy within the MHC-II in patients suffering from postoperative abdominal sepsis. Compared to a matched control cohort, we could demonstrate that postoperative sepsis was associated with a selective and persistent increase in CTCF enrichment at binding sites located next to classical HLA genes that code for proteins expressed on the surface of antigen-presenting cells. This was accompanied by a substantial decline in the transcription of the classical genes *HLA-DRA*, *HLA-DRB1*, *HLA-DPA1* and *HLA-DPB1* as well as the master regulator CIITA. Increased CTCF binding within the MHC-II persisted during the first week after sepsis diagnosis. In contrast, we observed a transcriptional recovery of CIITA that was associated with long-term survival. Overall, our findings underscore the relevance of CTCF mediated modulation of HLA class II gene expression in functionally impaired monocytes during postoperative abdominal sepsis.

In our collective, patients suffering from postoperative sepsis were characterized by high plasma levels of both pro-inflammatory IL-6 and anti-inflammatory IL-10 at the time of study inclusion, markers associated with disease severity and clinical outcome in sepsis [32, 33]. In contrast to a significant decrease in IL-6, plasma levels of IL-10 remained high during the study period, indicating a continuing shift of the immune response towards an immunosuppressive phenotype. In addition, we observed a strong decrease in cytokine production by blood cells of patients with postoperative sepsis after stimulation with LPS, which has repeatedly been described in inflammation and sepsis [34–38] as potential determinant of secondary infections in sepsis survivors [39].

Moreover, in our study, the extent of monocyte HLA-DR surface expression was already found to be diminished at the time when sepsis was diagnosed. The decrease of monocyte HLA-DR surface expression occurs early in sepsis [40], which supports the concept of timely counter-regulatory mechanisms that are initiated even before sepsis becomes clinically apparent [41]. Persisting low levels of HLA-DR molecules on monocyte surface, as seen in our study cohort, have previously been identified as independent predictor of secondary infections and mortality in patients with sepsis and septic shock [5, 7, 42].

On the cellular level, the observed drop of HLA-DR expression on monocyte surface in patients with postoperative sepsis indicates a loss of monocyte functionality in case of pathogen recognition and antigen presentation. These processes are at least partially regulated on the level of gene transcription [14], with CIITA playing an important role in the interaction with DNA-binding factors such as the CAMP Responsive Element Binding Protein (CREB), the Regulatory Factor X (RFX) or the Nuclear Transcription Factor Y (NFY) in MHC promoter regions [43, 44]. The significance of the master regulator for MHC expression has been accentuated by previous studies linking changes in CIITA expression to the pathogenesis of immune disorders or cancer [45, 46]. In line, we and others could demonstrate that reduced *CIITA* transcription during sepsis is accompanied by decreased HLA-DR mRNA levels as well as a reduced monocyte HLA-DR receptor density [18, 40]. However, we found that reduced *CIITA* transcription in patients with postoperative sepsis was not accompanied with a general HLA class II downregulation. Instead, postoperative sepsis was associated with selective changes in the expression of HLA class II genes coding for monocyte surface proteins. Even more importantly, the observed reduction in classical *HLA-DRA*, *HLA-DRB1*, *HLA-DPA1* and *HLA-DPB1* transcription as well as the decline in monocyte HLA-DR surface expression persisted in all patients with postoperative sepsis despite a recovery of *CIITA* transcription levels during the study period. Taken together, our findings support the idea of a much more complex regulatory network that modulates MHC transcription, possibly including the highly conserved zinc-finger protein CTCF.

Among the large number of potential target regions for CTCF spread across the human genome [19–21], Majumder and Boss identified ten binding sites inside the MHC-II region demarcating subregions that include HLA class II family genes (i.e. HLA-DP, HLA-DQ and HLA-DR genes) from those comprising non-HLA genes (i.e TAP or BRD) [22]. Remarkably, 3C-experiments using Raji cells—a B-cell line—revealed that the identified CTCF binding sites interact with selected, spatially related MHC-II promoter regions [22]. As an important finding, these interactions were confirmed for pairs of CTCF-sites and promoter regions of genes, which also displayed sepsis-induced alterations in our study (CM1 and HLA-DRA, CM2 and HLA-DRB1, CM9 and HLA-DPA1 and CM9 and HLA-DB1). This indicates a central link between CTCF-promoter-interaction and MHC gene transcription. Of note, no interactions between CTCF-binding sites and gene promoters were found in non-CIITA-expressing cell lines [22].

Remarkably, Majumder and colleagues also described a significantly reduced expression of classical MHC-II genes following CTCF knockdown in Raji-cells. Accordingly, the authors proposed a potential interaction between CTCF binding sites, CIITA, the RFX-CREB-NFY complex and MHC-II promoter regions requiring CTCF to achieve active gene transcription [16]. This is in line with own previous observations [18] as well as the findings in the present study, showing a basal occurrence of CTCF at almost all binding sites within the HLA-class II locus in monocytes of postoperative control patients that was accompanied by active HLA class II gene expression.

However, we observed an increased expression of CTCF as well as an inverse association between both CTCF expression and CTCF binding at selected sites within the HLA-class II locus and the expression of related HLA class II genes during postoperative sepsis. This is also in line with a previously conducted study of our group [18] and supports the assumption that the proposed interaction between CIITA, CTCF and its binding sites might be disturbed during critical illness like sepsis, resulting in a decoupled co-regulation of HLA genes.

Several mechanisms have been described to modulate CTCF occupancy [23], including the positioning of nucleosomes surrounding CTCF binding sites [47]. Since the acetylation of histones is generally associated with `open´ chromatin [48] and has previously been described in a cell culture model for a CTCF binding site within the MHC-II region [17], we analyzed the enrichment of H3K27ac at the investigated CTCF binding sites. However, beside a slight increase of histone acetylation at CM1, we did not observe a consistent impact of sepsis on H3K27ac enrichment at the investigated locus within MHC-II, indicating that other mechanisms modulate the differential CTCF binding during postoperative sepsis.

It is well known that epigenetic changes can endure [49], with even transgenerational effects such as alterations in DNA-methylation found in a murine model of sepsis survivors [50]. Although DNA-methylation especially at CpG sites of the genome represents another known determinant of CTCF occupancy [51], it is so far unknown whether CTCF binding itself is a long-lasting or even heritable epigenetic mark in human disease [23]. In our study, however, we could demonstrate that sepsis-associated increases in CTCF binding remain observable within 7 days within the MHC-II region of blood-derived CD14^+^ monocytes. With regard to the lifespan of circulating classical and intermediate CD14^+^ monocytes ranging between 1 and 4 days in healthy humans as well as the increased turnover of blood monocytes in a model of human endotoxemia [52], it is likely that the cells isolated in this study at T1 and T2 descended from different generations. Thus, although we cannot exclude that sepsis-associated stimuli persist and constantly induce CTCF-occupancy in peripheral blood monocytes, there is certain probability that sepsis might lead to endurable epigenetic changes. This includes altered CTCF binding in hematopoietic precursor cells as underlying cause of sepsis-induced immune alterations.

Ongoing impairment of the immune system is supposed to significantly contribute to the phenomenon of increased morbidity and delayed mortality after sepsis [53], the latter raising up to >40% after one year [54]. This is comparable to our collective of patients with postoperative sepsis. Interestingly, we detected a reconstitution of *CIITA* expression levels at T2 in those patients who survived sepsis within the first year after diagnosis. In previous work, we compared long-term survivors of sepsis with healthy control individuals matched for age, sex and comorbidities [55]. Interestingly, while the post-sepsis group was characterized by alterations in the expression of distinct pattern recognition receptors on the surface of circulating monocytes, no major differences between the groups were found concerning monocytic HLA-DR density. The fact that monocytic HLA-DR surface expression levels in long-term survivors are comparable to healthy controls, while septic patients in the present cohort still display significant alterations in HLA-DR surface expression as well as HLA class II gene expression at day 7 in our view hints towards a slow restoration process. Hence we speculate that CIITA recovery might serve is an early indicator for a recovery of immune function after sepsis.

There are several limitations in our study: First, we focused on one distinct cell type, monocytes, which is only one part in the complex sepsis-induced immune reaction. Furthermore, we exclusively investigated patients with postoperative abdominal sepsis. Therefore, our findings are not generalizable to other sepsis foci. Our study was conducted as a single center observation due to the necessity to immediately process the samples in the experimental workflow. Finally, as we only examined a comparably small number of patients in this pilot study, further investigations with larger sample sizes are needed to validate our findings.

In summary, by investigation of all known CTCF binding sites within the HLA class II locus, we discovered distinct sepsis-associated alterations in CTCF occupancy in circulating monocytes compared to a control cohort matched for type of surgery as well as age and gender—characteristics with potential impact on immune function. Differential CTCF enrichment persisted during the study period and was accompanied by distinct transcriptional alterations of spatially related classical HLA class II genes that are involved in monocyte surface presentation of microbial peptides to CD4^+^-T-lymphocytes. Taken together, these findings indicate an involvement of CTCF in modulating the transcriptional response of functionally impaired monocytes during the initial phase of postoperative human sepsis.

## Methods

### Ethics and patients

Experiments were conducted in accordance with the principles expressed in the Declaration of Helsinki and with the approval of the ethics committees of the medical faculty of Heidelberg University (Alte Glockengießerei 11/1, D-69115 Heidelberg, Germany; approval number S-135/2016). Written, informed consent was provided on forms approved by the Institutional Review Board either by the participants, or by their legal representatives. All participants were at least 18 years old. General exclusion criteria were pre-existing auto-immune diseases and / or immune-suppressive medication, pre-existing renal failure, infectious diseases (i.e. HIV, hepatitis) or the participation in an interventional study. This study is registered in the German Clinical Trial Register (Trial number: DRKS00011667).

Patients with sepsis due to an infectious abdominal complication following a major abdominal surgical intervention (i.e. due to anastomotic leakage) were included. Type of surgery and time to sepsis diagnosis were recorded. Sepsis had to be recognized within 24 hours prior to study inclusion according to international consensus definitions [3]. Blood samples were taken at the time of study inclusion (T1) and 7 days thereafter (T2) and were immediately processed in the experimental workflow.

Patients undergoing major abdominal surgery at our institution were screened for eligibility to participate as control patients by checking for general exclusion criteria and by matching for age, gender and type of intervention. To consider that possible postoperative complications including sepsis commonly occur several days after surgery, blood for experimental workflow was collected seven days after surgery to increase comparability with the sepsis cohort. The study design is visualized in Fig 1.

### Data collection and standard laboratory parameters

Socio-demographic and clinical data including pre-existing comorbidities based on the Charlson comorbidity index [56] were extracted from electronic and paper-based records. Standard laboratory parameters were measured in the routine hospital laboratory according to in-house standards.

### Ex vivo whole blood stimulation

To assess the functionality of circulating immune cells, whole blood was incubated for 24 hours with ultrapure lipopolysaccharide (LPS, Invivogen, San Diego, CA, USA) after 1:1 dilution with RPMI1640 containing proprietary GlutMAX^™^ (Thermo Fisher Scientific, Waltham, MA, USA) and 5% fetal bovine serum (Ultra-low endotoxin; Cell Concepts GmbH, Umkirch, Germany). Supernatant was recovered after centrifugation (5.000 rpm, 5 minutes) and used for subsequent enzyme-linked immunosorbent assay (ELISA).

### Enzyme-linked immunosorbent assay

Both, plasma derived from untreated, directly centrifuged whole blood as well as supernatants from *ex-vivo* stimulated samples were used for ELISA experiments to determine the concentration of cytokines (IL-6, IL-10) according to manufacturer`s instructions (R&D Technologies, North Kingstown, USA).

### Monocyte surface expression of HLA-DR

To quantify the expression of HLA-DR on monocyte surface, flow cytometry (FACSVerse^™^ flow cytometer, BD Bioscience, Heidelberg, Germany) was conducted after incubating 50 μl ETDA-anti-coagulated whole blood with 20 μl anti-HLA-DR antibody (Quantibrite anti-HLA-DR/Monocyte antibody, BD Bioscience, Heidelberg, Germany) and after lysis of erythrocytes (FACS Lysing solution BD Bioscience, Heidelberg, Germany) as previously described and according to manufacturer`s instructions [18]. HLA-DR surface expression was measured as median fluorescence intensity after gating out monocytes based on CD14 expression (S10 Fig).

For quantification of HLA-DR molecules on cell surface (`counts per monocyte´), determined sample values were converted using daily measured 4-point calibration curves (Quantibrite PE Beads, BD Bioscience, Heidelberg, Germany).

### Monocyte separation

Monocytes were isolated from a 30 ml Lithium-heparin-anti-coagulated blood sample in accordance to a previously described protocol [57]. In particular, Ficoll-based density gradient centrifugation to extract peripheral blood mononuclear cells was followed by magnetic cell sorting (autoMACS, Miltenyi Biotec, Bergisch Gladbach, Germany) using CD14 MicroBeads (Miltenyi Biotec, Bergisch Gladbach, Germany). Purity of the isolated monocytes was ensured by flow cytometry (FACSVerse^™^ flow cytometer, BD Bioscience, Heidelberg, Germany) using an anti-human-CD14-FITC antibody (BioLegend, San Diego, USA).

### Gene expression analysis

A column-based method (RNeasy Plus Mini Kit, Qiagen, Hilden, Germany) allowed extraction of RNA from isolated monocytes for gene expression analysis. Spectrophotometry (Nanodrop, Thermo Fisher Scientific, Waltham, USA) was performed to confirm sufficient concentration and quality of the extracted RNA. All RNA samples showed 260/280 as well as 260/230 ratios above 1.8. For subsequent reverse transcription, 1 μg RNA was processed according to manufacturer`s instructions (Quantitect Reverse Transcription Kit, Qiagen, Hilden, Germany). Commercial TaqMan^™^ assays (Applied Biosystems, Foster City, USA; see **Table A in** S4 Table) and reagents (TaqMan^™^ Gene Expression Master Mix, Applied Biosystems, Foster City, USA) were used for subsequent quantitative PCR experiments on a StepOnePlusTM PCR-cycler (Applied Biosystems, Foster City, USA).

All reactions were done in triplicate and mean Ct values were calculated for each gene. For ΔCt analysis the values of the gene of interest were subtracted from the mean of the two endogenous control genes *Actin Beta* (ACTB) and *Hypoxanthine Phosphoribosyltransferase 1* (HPRT1). Relative gene expression was determined by 2^ΔCt^.

### Chromatin immunoprecipitation and quantitative PCR analysis

Before chromatin immunoprecipitation (ChIP), isolated monocytes were cross-linked for 10 minutes at 18°C using formaldehyde at a final concentration of 1%. Fixation of DNA-protein links was stopped with 0.125 M glycine. After cell lysis (10^6^ cells per 200 μl lysis buffer), DNA-shearing was conducted in the Bioruptor Pico^®^ (Diagenode, Liège, Belgium) to achieve fragment sizes of 150–200 base pairs. Immunoprecipitation, reverse cross-linking and DNA purification were performed using an automated system (IP-Star^®^ Compact) with specific reagent kits (Auto iDeal ChIP-seq Kit for Transcription Factors, Auto iDeal ChIP-seq Kit for Histones) as well as antibodies against CTCF, trimethylated lysine 27 of histone 3 (H3K4me3) and acetylated lysine 27 of histone 3 (H3K27ac, all obtained from Diagenode, Liège, Belgium) according to manufacturer`s instructions. The amount of obtained ChIP-DNA (diluted in pure H_2_O) was quantified using a Qubit^™^ assay (Qubit Fluorometric Quantitation, Thermo Fisher Scientific, Waltham, USA). Subsequent quantitative PCR analysis was done using a StepOnePlusTM PCR-cycler (Applied Biosystems, Foster City, USA) as well as specific primer sets for CM 1–10 as previously identified by Majumder and Boss [22] or primer sets for the promoter regions of classical HLA class II genes. ChIP-antibodies and primer sequences are listed in **Tables B and C in** S4 Table.

### Statistical analysis and data visualization

Raw data for all figures are provided in S5 Table. Results were visualized as mean and standard error of the mean (SEM) and analyzed using GraphPad Prism (Version 6.0f, GraphPad Software, La Jolla, USA). Comparisons were performed using Fisher’s exact test for categorical data and Mann-Whitney U test for continuous data. Spearman test was conducted for correlation analysis. P values <0.05 were considered significant and indicated with ‘*’ (p<0.05, >0.01), ‘**’ (p<0.01), ‘***’ (p<0.001) or ‘****’ (p<0.0001).

## Supporting information

S1 Table Socio-demographic and clinical characteristics of the study population. (XLSX)

S2 Table Correlation between the expression of classical HLA genes, *CIITA* and *CTCF*. (XLSX)

S3 Table Laboratory parameters associated with sepsis and cytokine levels at T1 and T2. (XLSX)

S4 Table Table A: TaqManTM assays. Table B: ChIP antibodies. Table C: ChIP-qPCR primers.
(XLSX)

S5 Table Raw data. (XLSX)

S1 Fig CTCF and H3K27ac occupancy at specific control regions outside the MHC-II region. Chromatin from isolated human CD14^++^ monocytes (control patients, sepsis patients at the time of sepsis diagnosis, T1, and 7 days thereafter) was immunoprecipitated with **(A)** anti-CTCF antibody, **(B)** anti-H3K27ac and **(C)** anti-H3K4me3 antibodies for subsequent qPCR using primer pairs selected genome regions serving as positive and negative controls. H19-ICR (located on chromosome 11) served as positive control for CTCF-binding. *MB* (located on chromosome 22) served as negative control for CTCF binding and H3K4me3 and *EIF4A2* (located on chromosome 3) as positive control for H3K27ac and H3K4me3. *MYT1* (located on chromosome 20) was used as negative control for H3K27ac. Mann-Whitney U test, p>0.05, control patients n = 9 (A and B), n = 8 (C); patients with sepsis n = 9 (A and B), n = 8 (C); mean ± SEM).
(TIF)

S2 Fig CTCF occupancy at specific binding sites within the MHC-II region. Chromatin from isolated human CD14^++^ monocytes was immunoprecipitated with anti-CTCF antibody for subsequent qPCR using primer pairs on CTCF binding sites within the investigated MHC-II region **(A)** CM4, **(B)** CM5, **(C)** CM6, **(D)** CM7, **(E)** CM8 and **(F)** CM10; all p>0.05, Mann-Whitney U test, control patients n = 9, patients with sepsis at the time of sepsis diagnosis (T1) n = 9, mean ± SEM.
(TIF)

S3 Fig Level of histone acetylation at CTCF binding sites within the MHC-II region. Chromatin from isolated human CD14^+^ monocytes was immunoprecipitated with anti-H3K27ac antibody for subsequent qPCR using primer pairs on CTCF binding sites within MHC-II region. **(A)** CM1 (p<0.05, Mann-Whitney U test, control patients n = 9, patients with sepsis at the time of sepsis diagnosis (T1) n = 9, mean ± SEM), **(B)** CM2 (p>0.05, Mann-Whitney U test, control patients n = 9, patients with sepsis at the time of sepsis diagnosis (T1) n = 9, mean ± SEM), **(C)** CM3 (p>0.05, Mann-Whitney U test, control patients n = 9, patients with sepsis at the time of sepsis diagnosis (T1) n = 9, mean ± SEM) and **(D)** CM9 (p>0.05, Mann-Whitney U test, control patients n = 9, patients with sepsis at the time of sepsis diagnosis (T1) n = 9, mean ± SEM).
(TIF)

S4 Fig Expression of HLA class II genes encoding for different HLA isotypes. RNA from isolated human CD14^++^ monocytes was used for reverse transcription and subsequent qPCR experiments using TaqMan Assay against **(A)** HLA-DRB3, **(B)** HLA-DRB5, **(C)** HLA-DQA1, **(D)** HLA-DQB1, **(E)** HLA-DQA2 and **(F)** HLA-DQB2 (all p>0.05, Mann-Whitney U test, control patients n = 9, patients with sepsis at the time of sepsis diagnosis (T1) n = 9, mean ± SEM).
(TIF)

S5 Fig H3K4me3 levels in the promoter regions of classical HLA genes. Chromatin from isolated human CD14^+^ monocytes was immunoprecipitated with anti-H3K4me3 antibody for subsequent qPCR using primer pairs on promoter regions of **(A)** HLA-DRA, **(B)** HLA-DRB1, **(C)** HLA-DPA1 and **(D)** HLA-DPB1 (all p<0.05, Mann-Whitney U test, control patients n = 8, patients with sepsis at the time of sepsis diagnosis (T1) n = 8, mean ± SEM).
(TIF)

S6 Fig HLA-DR expression on monocyte surface during the time course of sepsis. Surface expression of HLA-DR on CD14^+^-monocytes (patients with sepsis at the time of sepsis diagnosis (T1) and 7 days thereafter (T2)) was analyzed in whole blood samples using flow cytometry and is compared between T1 and T2 as well as between survivors and non-survivors (all p>0.05, Mann-Whitney U test, patients with sepsis n = 9, survivors n = 5, non-survivors n = 4, mean ± SEM).
(TIF)

S7 Fig Expression of CTCF during the time course of sepsis. RNA from isolated human CD14^++^ monocytes (patients with sepsis at the time of sepsis diagnosis (T1) and 7 days thereafter (T2)) was used for reverse transcription and subsequent qPCR experiments using TaqMan Assay against CTCF. Relative gene expression is compared between T1 and T2 as well as between survivors and non-survivors (all p>0.05, Mann-Whitney U test, patients with sepsis n = 9, survivors n = 5, non-survivors n = 4, mean ± SEM).
(TIF)

S8 Fig CTCF occupancy at specific binding sites within the MHC-II region during the time course of sepsis. Chromatin from isolated human CD14^++^ monocytes (patients with sepsis at the time of sepsis diagnosis (T1) and 7 days thereafter (T2)) was immunoprecipitated with anti-CTCF antibody for subsequent qPCR using primer pairs on specific target regions within the MHC-II region. Enrichment of CTCF at the binding sites **(A)** CM1, **(B)** CM2, **(C)** CM3 and **(D)** CM9 is shown and compared between T1 and T2 as well as between survivors and non-survivors (all p>0.05, Mann-Whitney U test, patients with sepsis n = 9, survivors n = 5, non-survivors n = 4, mean ± SEM).
(TIF)

S9 Fig Expression of classical MHC-II genes during the time course of sepsis. RNA from isolated human CD14+ monocytes (patients with sepsis at the time of sepsis diagnosis (T1) and 7 days thereafter (T2)) was used for reverse transcription and subsequent qPCR experiments using TaqMan Assay against **(A)** HLA-DRA, **(B)** HLA-DRB1, **(C)** HLA-DPA1 and **(D)** HLA-DPB1. Relative gene expression is compared between T1 and T2 as well as between survivors and non-survivors (all p>0.05, Mann-Whitney U test, patients with sepsis n = 9, survivors n = 5, non-survivors n = 4, mean ± SEM).
(TIF)

S10 Fig Gating strategy. (TIF)
