# Supplementary figures and images for: Postoperative abdominal sepsis induces selective and persistent changes in CTCF binding within the MHC-II region of human monocytes
Source: PLoS One. 2021 May 3;16(5):e0250818. doi: 10.1371/journal.pone.0250818 (PMC8092803; doi:10.1371/journal.pone.0250818)

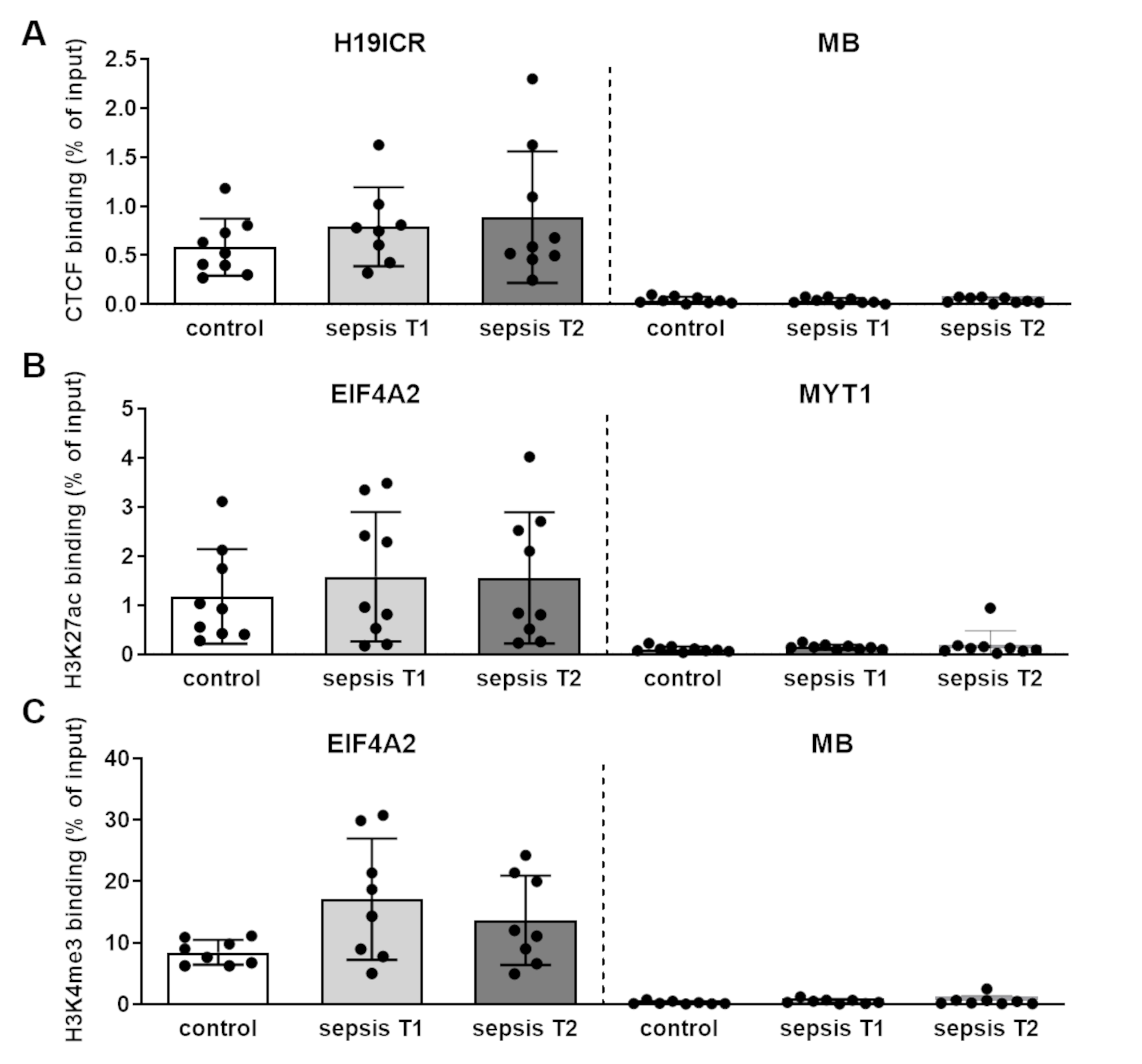

Supplement: S1 Fig — Chromatin from isolated human CD14++ monocytes (control patients, sepsis patients at the time of sepsis diagnosis, T1, and 7 days thereafter) was immunoprecipitated with (A) anti-CTCF antibody, (B) anti-H3K27ac and (C) anti-H3K4me3 antibodies for subsequent qPCR using primer pairs selected genome regions serving as positive and negative controls. H19-ICR (located on chromosome 11) served as positive control for CTCF-binding. MB (located on chromosome 22) served as negative control for CTCF binding and H3K4me3 and EIF4A2 (located on chromosome 3) as positive control for H3K27ac and H3K4me3. MYT1 (located on chromosome 20) was used as negative control for H3K27ac. Mann-Whitney U test, p>0.05, control patients n = 9 (A and B), n = 8 (C); patients with sepsis n = 9 (A and B), n = 8 (C); mean ± SEM). (TIF) [file pone.0250818.s006.tif]

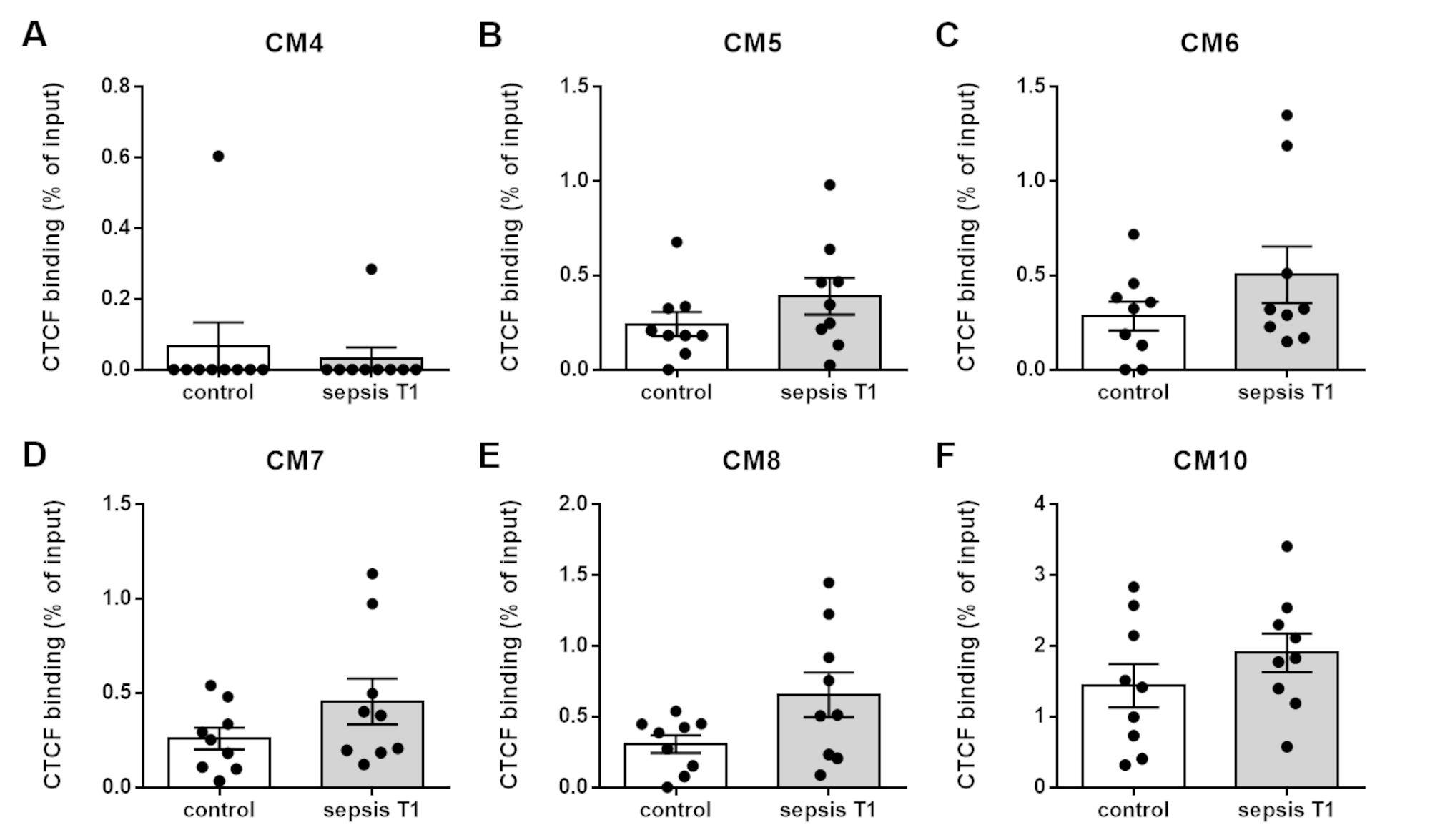

Supplement: S2 Fig — Chromatin from isolated human CD14++ monocytes was immunoprecipitated with anti-CTCF antibody for subsequent qPCR using primer pairs on CTCF binding sites within the investigated MHC-II region (A) CM4, (B) CM5, (C) CM6, (D) CM7, (E) CM8 and (F) CM10; all p>0.05, Mann-Whitney U test, control patients n = 9, patients with sepsis at the time of sepsis diagnosis (T1) n = 9, mean ± SEM. (TIF) [file pone.0250818.s007.tif]

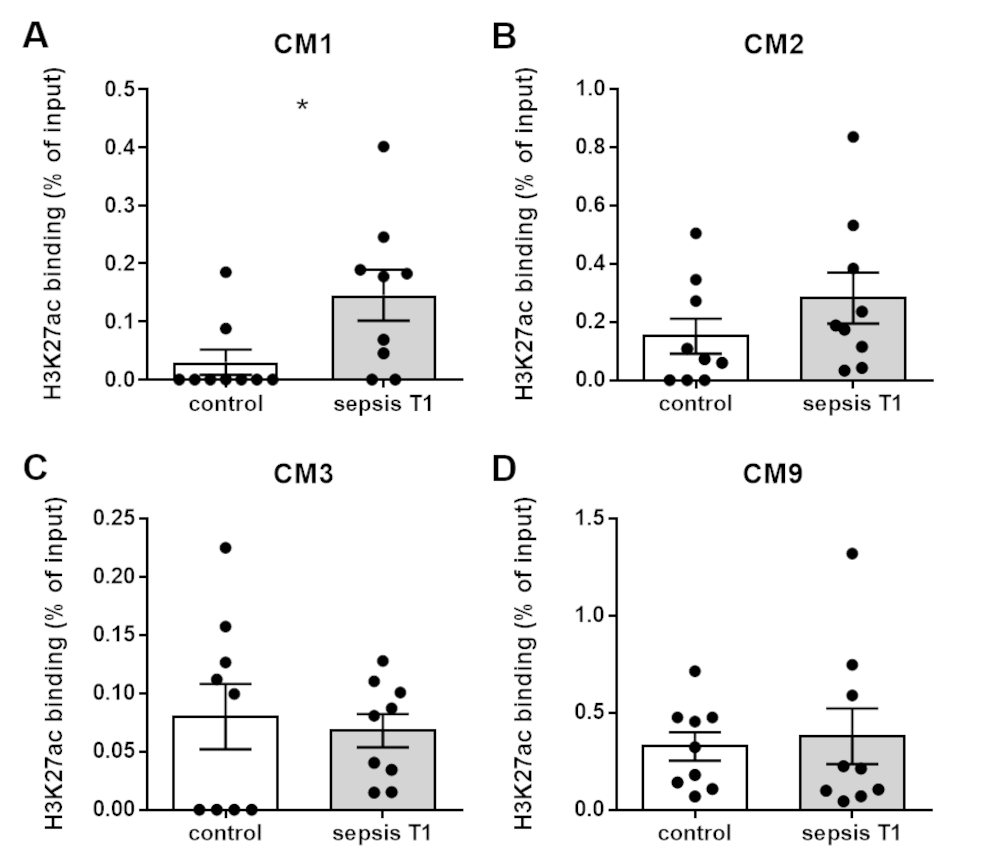

Supplement: S3 Fig — Chromatin from isolated human CD14+ monocytes was immunoprecipitated with anti-H3K27ac antibody for subsequent qPCR using primer pairs on CTCF binding sites within MHC-II region. (A) CM1 (p<0.05, Mann-Whitney U test, control patients n = 9, patients with sepsis at the time of sepsis diagnosis (T1) n = 9, mean ± SEM), (B) CM2 (p>0.05, Mann-Whitney U test, control patients n = 9, patients with sepsis at the time of sepsis diagnosis (T1) n = 9, mean ± SEM), (C) CM3 (p>0.05, Mann-Whitney U test, control patients n = 9, patients with sepsis at the time of sepsis diagnosis (T1) n = 9, mean ± SEM) and (D) CM9 (p>0.05, Mann-Whitney U test, control patients n = 9, patients with sepsis at the time of sepsis diagnosis (T1) n = 9, mean ± SEM). (TIF) [file pone.0250818.s008.tif]

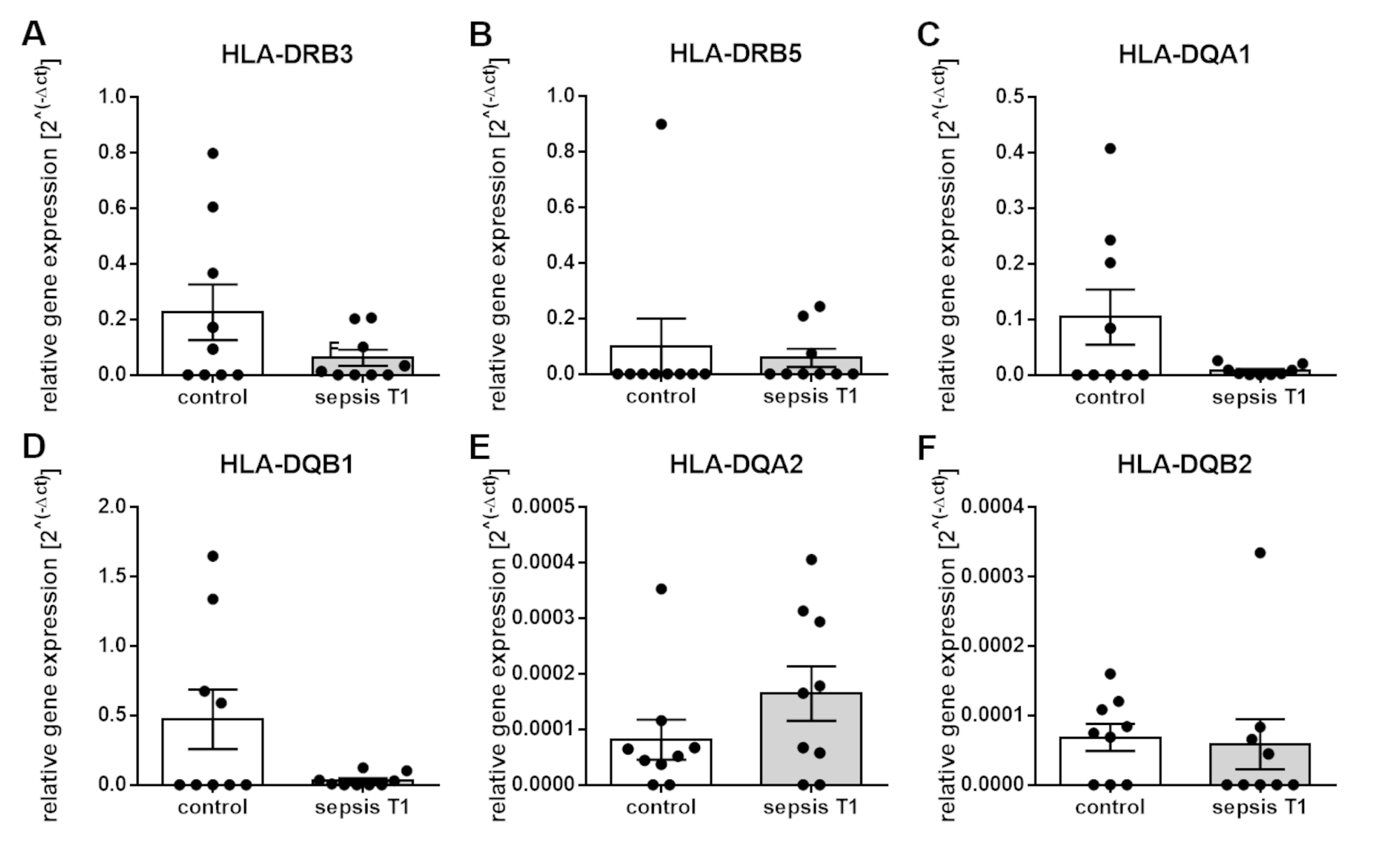

Supplement: S4 Fig — RNA from isolated human CD14++ monocytes was used for reverse transcription and subsequent qPCR experiments using TaqMan Assay against (A) HLA-DRB3, (B) HLA-DRB5, (C) HLA-DQA1, (D) HLA-DQB1, (E) HLA-DQA2 and (F) HLA-DQB2 (all p>0.05, Mann-Whitney U test, control patients n = 9, patients with sepsis at the time of sepsis diagnosis (T1) n = 9, mean ± SEM). (TIF) [file pone.0250818.s009.tif]

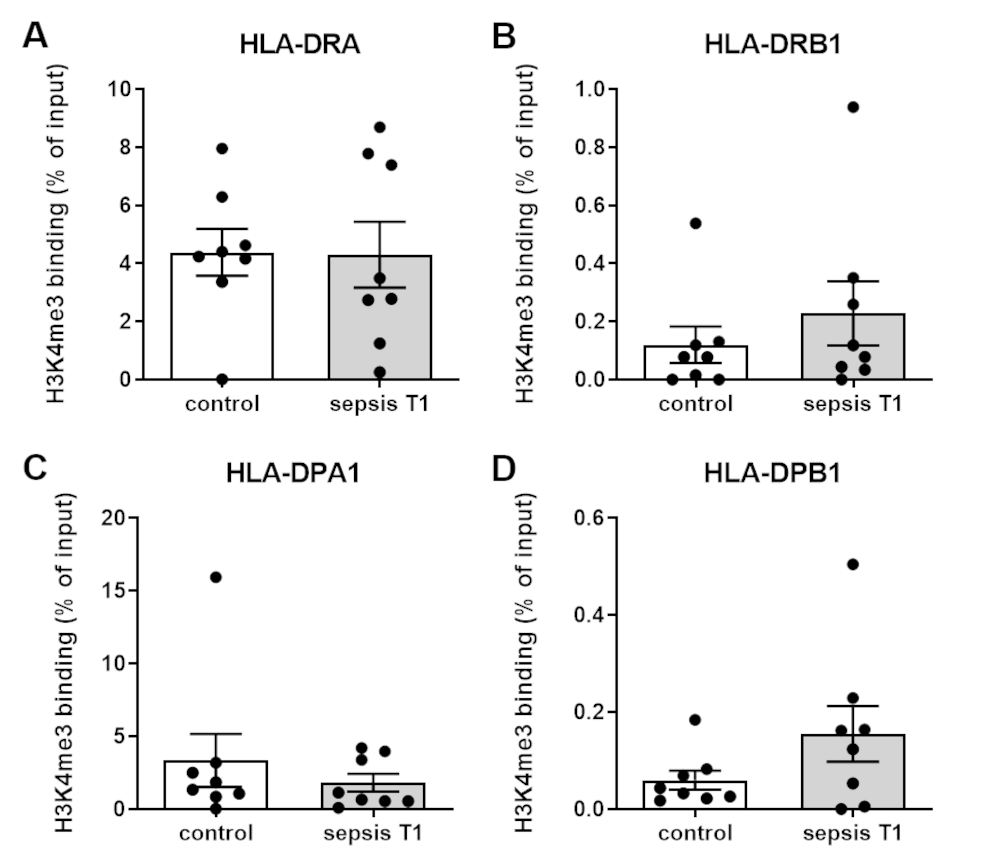

Supplement: S5 Fig — Chromatin from isolated human CD14+ monocytes was immunoprecipitated with anti-H3K4me3 antibody for subsequent qPCR using primer pairs on promoter regions of (A) HLA-DRA, (B) HLA-DRB1, (C) HLA-DPA1 and (D) HLA-DPB1 (all p<0.05, Mann-Whitney U test, control patients n = 8, patients with sepsis at the time of sepsis diagnosis (T1) n = 8, mean ± SEM). (TIF) [file pone.0250818.s010.tif]

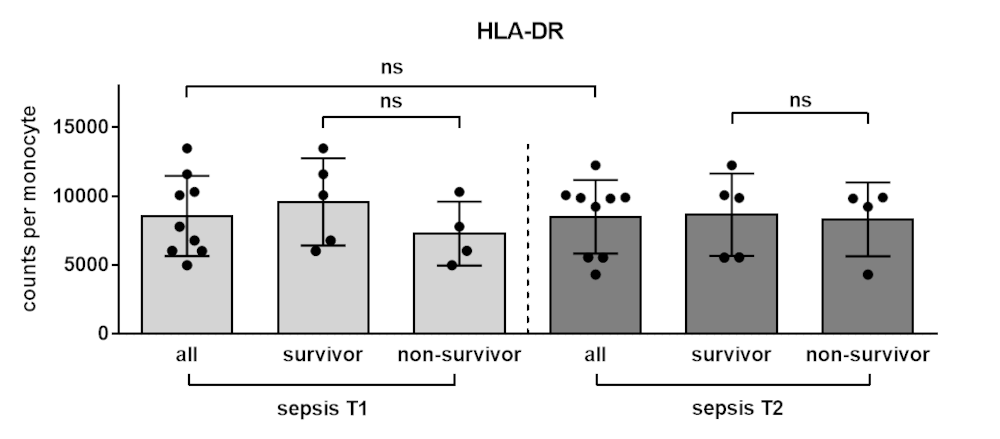

Supplement: S6 Fig — Surface expression of HLA-DR on CD14+-monocytes (patients with sepsis at the time of sepsis diagnosis (T1) and 7 days thereafter (T2)) was analyzed in whole blood samples using flow cytometry and is compared between T1 and T2 as well as between survivors and non-survivors (all p>0.05, Mann-Whitney U test, patients with sepsis n = 9, survivors n = 5, non-survivors n = 4, mean ± SEM). (TIF) [file pone.0250818.s011.tif]

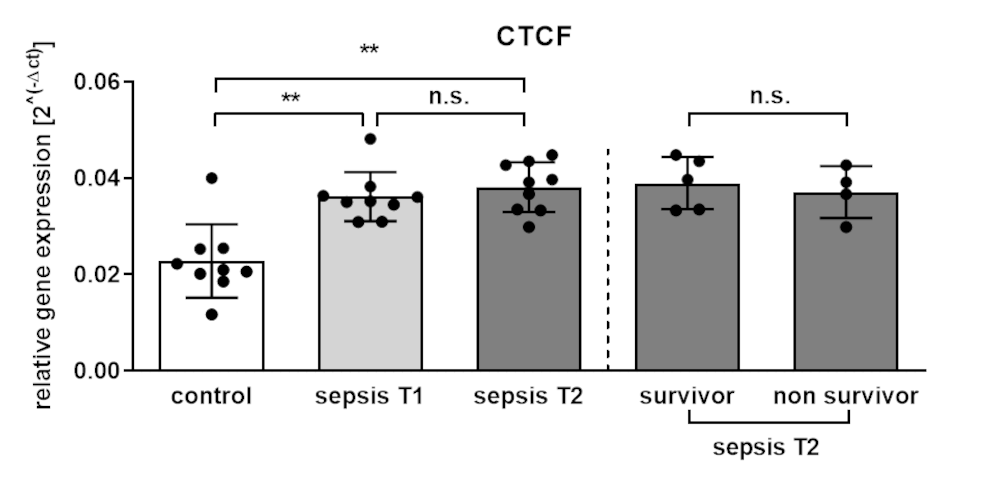

Supplement: S7 Fig — RNA from isolated human CD14++ monocytes (patients with sepsis at the time of sepsis diagnosis (T1) and 7 days thereafter (T2)) was used for reverse transcription and subsequent qPCR experiments using TaqMan Assay against CTCF. Relative gene expression is compared between T1 and T2 as well as between survivors and non-survivors (all p>0.05, Mann-Whitney U test, patients with sepsis n = 9, survivors n = 5, non-survivors n = 4, mean ± SEM). (TIF) [file pone.0250818.s012.tif]

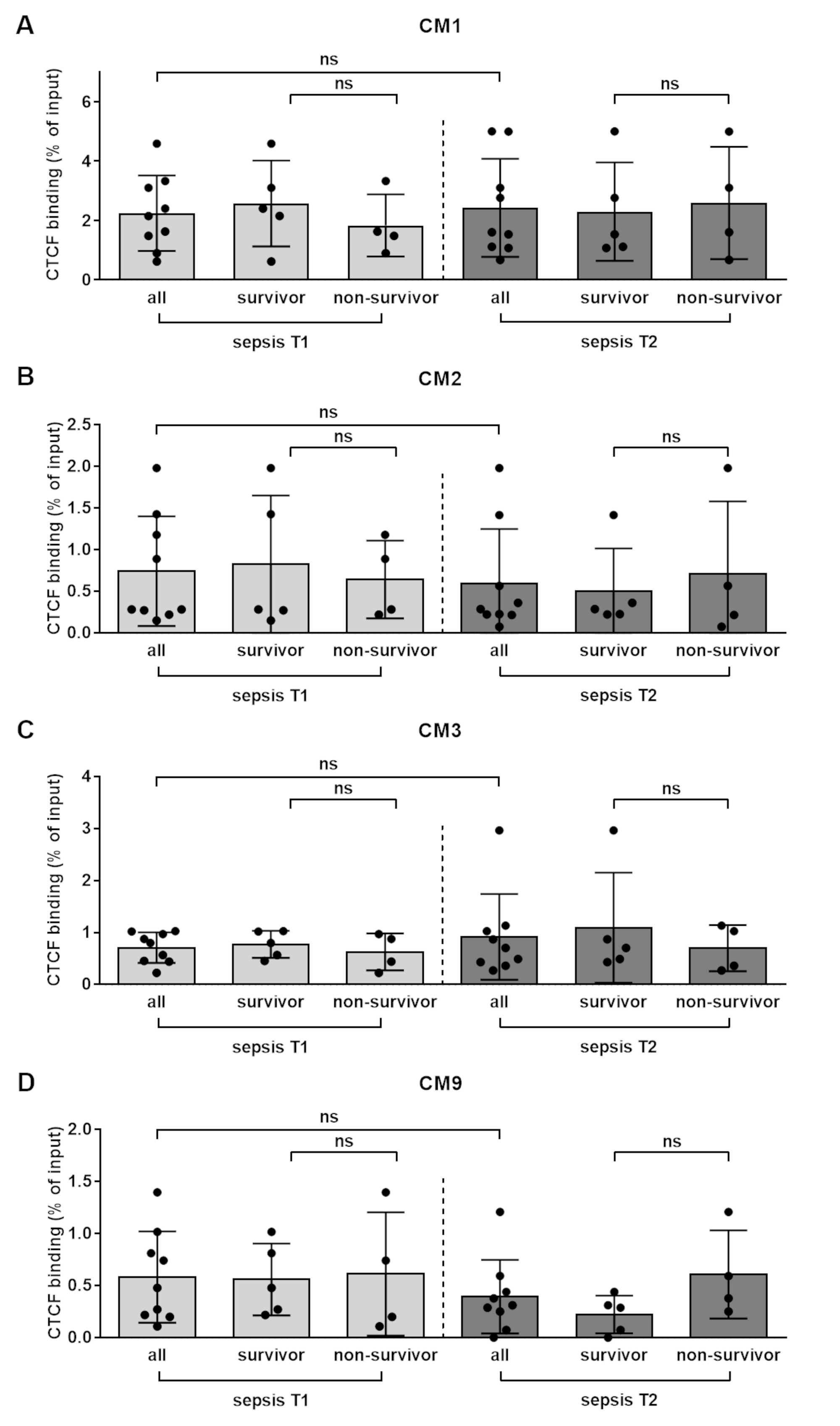

Supplement: S8 Fig — Chromatin from isolated human CD14++ monocytes (patients with sepsis at the time of sepsis diagnosis (T1) and 7 days thereafter (T2)) was immunoprecipitated with anti-CTCF antibody for subsequent qPCR using primer pairs on specific target regions within the MHC-II region. Enrichment of CTCF at the binding sites (A) CM1, (B) CM2, (C) CM3 and (D) CM9 is shown and compared between T1 and T2 as well as between survivors and non-survivors (all p>0.05, Mann-Whitney U test, patients with sepsis n = 9, survivors n = 5, non-survivors n = 4, mean ± SEM). (TIF) [file pone.0250818.s013.tif]

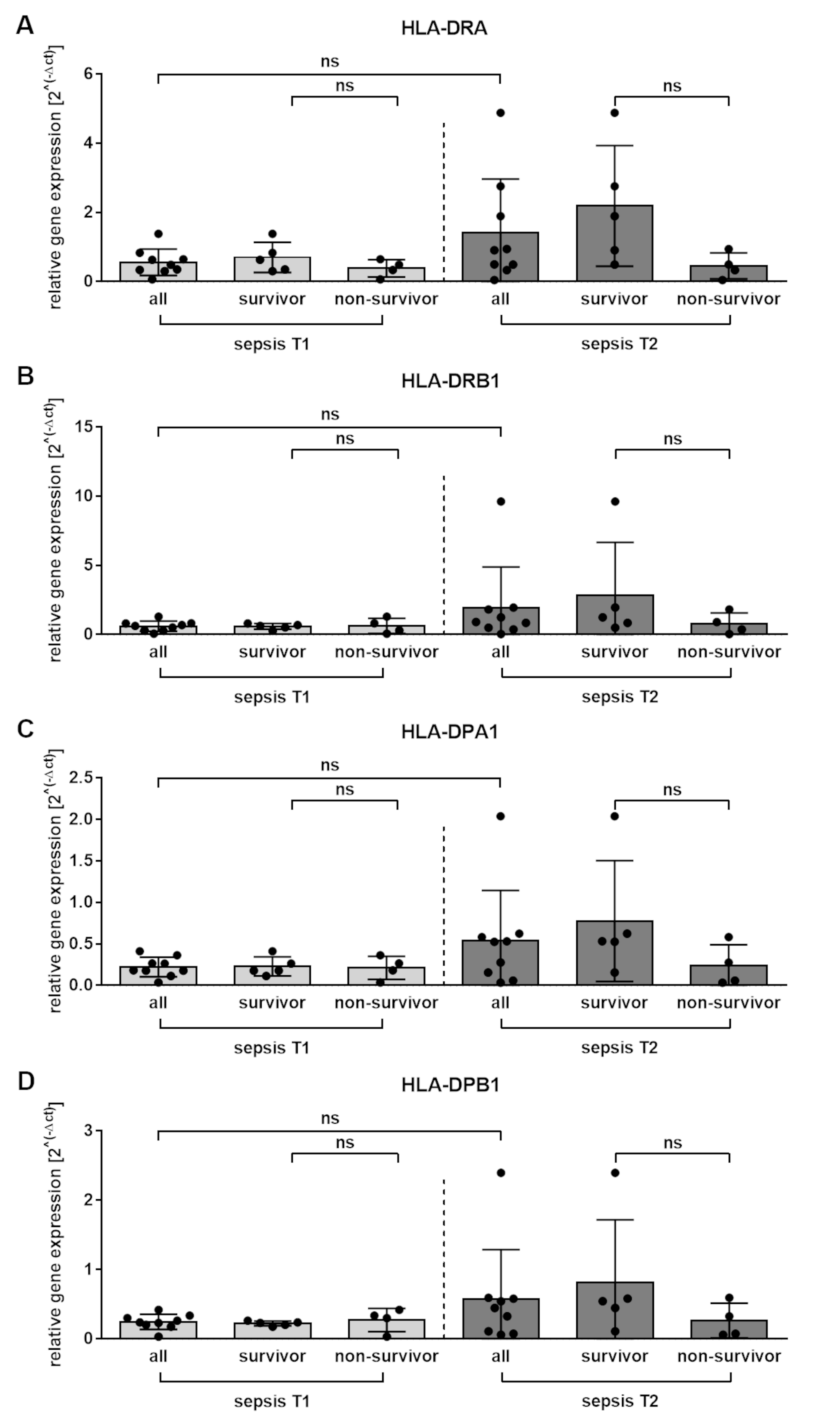

Supplement: S9 Fig — RNA from isolated human CD14+ monocytes (patients with sepsis at the time of sepsis diagnosis (T1) and 7 days thereafter (T2)) was used for reverse transcription and subsequent qPCR experiments using TaqMan Assay against (A) HLA-DRA, (B) HLA-DRB1, (C) HLA-DPA1 and (D) HLA-DPB1. Relative gene expression is compared between T1 and T2 as well as between survivors and non-survivors (all p>0.05, Mann-Whitney U test, patients with sepsis n = 9, survivors n = 5, non-survivors n = 4, mean ± SEM). (TIF) [file pone.0250818.s014.tif]

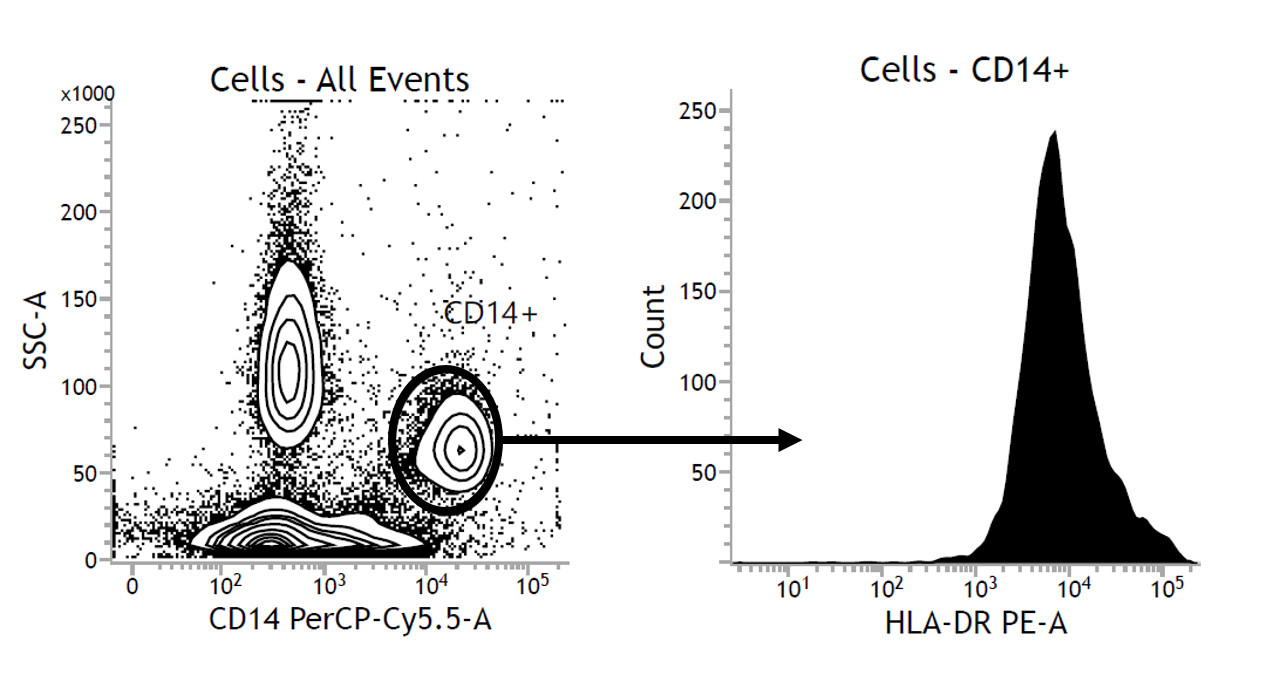

Supplement: S10 Fig — (TIF) [file pone.0250818.s015.tif]
